# Low Phospholipid‐Associated Cholelithiasis Syndrome: A Case Report of Chronic Recurrent Cholangitis Post‐Cholecystectomy Course

**DOI:** 10.1002/ccr3.70942

**Published:** 2025-09-24

**Authors:** Faris Bandar Alrashdan, Ramy Mahmoud Fathy Elbarody, Atheer Abdullah Ali Aldra, Mahmoud Salah Abdalhakeem Mohammed, Abdulaziz Althafery, Haidara Bohsas, Bisher Sawaf, Nashaat Kamal Hamdy Elkalagi

**Affiliations:** ^1^ Head of Gastroenterology Department King Salman Specialist Hospital, Hail Health Cluster Hail Saudi Arabia; ^2^ Gastroenterology Department King Salman Specialist Hospital, Hail Health Cluster Hail Saudi Arabia; ^3^ King Salman Specialist Hospital Hail Hail Saudi Arabia; ^4^ Faculty of Medicine Aleppo University Aleppo Syria; ^5^ Department of Internal Medicine University of Toledo Medical Center Toledo Ohio USA; ^6^ Department of Internal Medicine, Faculty of Medicine Arish University Arish Egypt

**Keywords:** cholangitis, cholecystectomy, chronic recurrent cholangitis, gallstone disease, low phospholipid‐associated cholelithiasis

## Abstract

Low phospholipid‐associated cholelithiasis (LPAC) syndrome is a rare and underdiagnosed cause of recurrent intrahepatic and extrahepatic bile duct stones. We report the case of a 32‐year‐old male with a history of laparoscopic cholecystectomy and multiple ERCPs who presented with acute epigastric pain and vomiting. He had recurrent episodes of cholangitis over several months, each managed with ERCP, balloon extraction, and biliary stenting. Imaging revealed biliary dilatation with obstructive stones. Emergency ERCP retrieved a large stone with pus and debris, followed by stent placement and clinical improvement. He was diagnosed with LPAC syndrome and discharged on ursodeoxycholic acid. Follow‐up imaging demonstrated persistent but improving biliary dilation without structural anomalies or abscesses. This case underscores the recurrent nature of LPAC syndrome and the importance of considering this diagnosis in young adults with unexplained biliary symptoms post‐cholecystectomy. Early recognition and long‐term management are essential to reduce recurrence.

**Summary**

- Low phospholipid‐associated cholelithiasis (LPAC) syndrome should be considered in young adults with recurrent biliary symptoms post‐cholecystectomy.
- Early diagnosis and long‐term medical management, such as ursodeoxycholic acid, are crucial to prevent recurrence and avoid repeated invasive procedures.

## 1 Introduction

Low phospholipid‐associated cholelithiasis (LPAC) syndrome is an uncommon hereditary condition resulting from variants of the ATP‐binding cassette sub‐family B member 4 (ABCB4/MDR3) gene. These genetic variants disrupt the secretion of biliary phosphatidylcholine, leading to cholesterol supersaturation in bile and the subsequent formation of numerous cholesterol gallstones in both the gallbladder and intrahepatic bile ducts [1]. LPAC constitutes a rare form of gallstone disease, representing roughly 1% of adults with symptomatic cholelithiasis [2]. Gallstones affect up to 10% of Europeans and Americans, with approximately 25% symptomatic and fewer than 2% developing severe complications such as cholangitis or pancreatitis [3]. Clinically, LPAC often occurs in young adults, usually under 40 years of age, presenting with recurring episodes of biliary colic or complications like cholangitis and pancreatitis, often without the presence of usual risk factors such as obesity [1]. A distinguishing feature of LPAC is the persistence of biliary symptoms after cholecystectomy, posing a diagnostic challenge and setting it apart from more common gallstone diseases [1]. The condition is often not adequately recognized due to insufficient awareness among healthcare providers and its recurrent nature, necessitating a heightened level of suspicion for accurate diagnosis [1]. Prolonged treatment with ursodeoxycholic acid (UDCA) is generally needed, as cholecystectomy alone is inadequate to prevent recurrence [4]. Due to the rarity of LPAC and its propensity for recurring biliary complications, it should be considered in patients presenting with unexplained chronic cholangitis after cholecystectomy. This case is presented to emphasize the diagnostic and therapeutic difficulties encountered in managing LPAC syndrome in a patient with chronic recurrent cholangitis after cholecystectomy.

## 2 Case History/Examination

A 32‐year‐old male presented with a 2‐day history of epigastric pain radiating to the back, accompanied by vomiting. He denied fever, chills, rigors, pruritus, or changes in urine or stool color. He was a non‐smoker with no history of alcohol consumption, herbal medication use, diabetes mellitus, or hypertension. His surgical history included a laparoscopic cholecystectomy 5 years ago, reportedly preceded by ERCP with common bile duct (CBD) stent placement, and a laparoscopic appendectomy 1 year prior.

He had undergone multiple prior ERCP procedures for CBD stone extraction and stent placements. Two MRCPs showed intrahepatic and extrahepatic bile duct dilatation with stones. Seven months prior, he presented with acute cholangitis, including abdominal pain, jaundice, fever, and rigors. ERCP was performed for stent removal, draining pus and multiple stones. Two weeks later, he developed another episode of cholangitis with choledocholithiasis. Repeat ERCP showed intrahepatic and extrahepatic ductal dilatation and stones. Balloon sweeping cleared the duct with drainage of clear bile, and a new stent was inserted.

## 3 Differential Diagnosis, Investigations and Treatment

The patient was referred to a tertiary hepatobiliary center for further evaluation and genetic counseling. There, sequential ERCPs were done to remove and replace the CBD stents. During the current admission, abdominal ultrasound showed moderate CBD dilatation and two small stones in the distal common hepatic duct. On examination, he was hypotensive, tachycardic, and had epigastric tenderness. The abdomen was soft and non‐distended, with no peritonitis, organomegaly, or ascites. ECG and chest X‐ray were unremarkable.

Initial considerations included recurrent cholangitis, residual CBD stones, biliary sludge, post‐cholecystectomy syndrome, or a rare biliary disorder.

Lab investigations showed elevated WBC count (16.52 × 10^3^/μL), CRP (16.6 mg/dL), GGT (375 U/L), ALP (237 U/L), total bilirubin (93 μmol/L), conjugated bilirubin (70 μmol/L), ALT (484 U/L), and AST (276 U/L).

**FIGURE 1:**
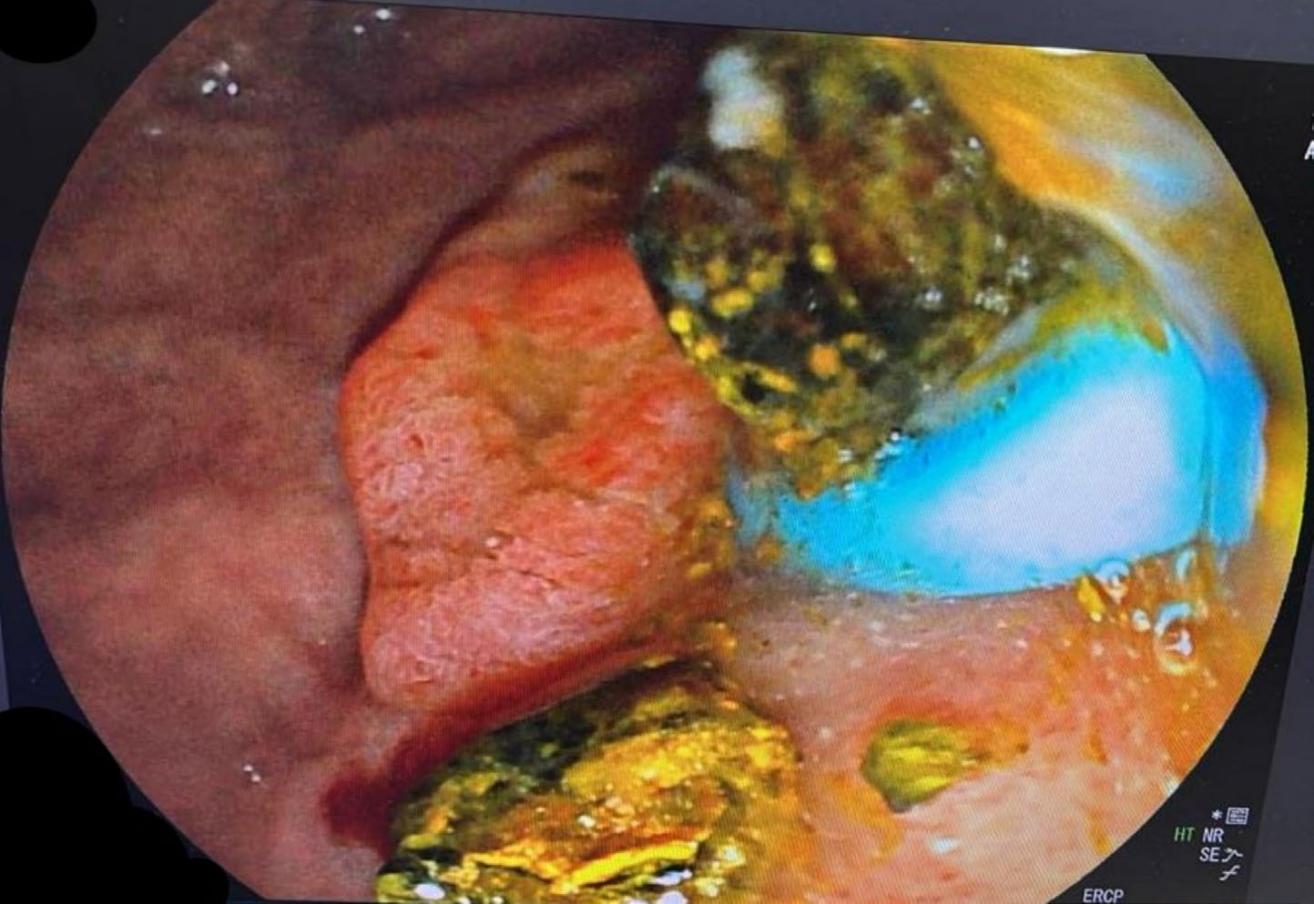
ERCP image showing large CBD stones, pus, and debris extracted during intervention for acute cholangitis and septic shock. A plastic stent was placed after duct clearance (The image was edited by Paint 3D to hide the personal details of the patient).

An urgent ERCP was performed for suspected acute cholangitis and septic shock. Selective CBD cannulation revealed proximal CBD filling defects. Multiple balloon sweeps (9–12 mm) removed pus, sludge, and debris, including a 1 × 2 cm stone. Occlusion cholangiogram confirmed ductal clearance. Pus was sent for culture. A 10F × 10 cm plastic CBD stent was inserted, and bile drainage was adequate. (Figure 1) The patient was treated with IV antibiotics for 1 week, with clinical and laboratory improvement. He was diagnosed with low phospholipid‐associated cholelithiasis (LPAC) syndrome and discharged on ursodeoxycholic acid (UDCA).

## 4 Conclusion and Results (Outcome and Follow‐Up)

**FIGURE 2:**
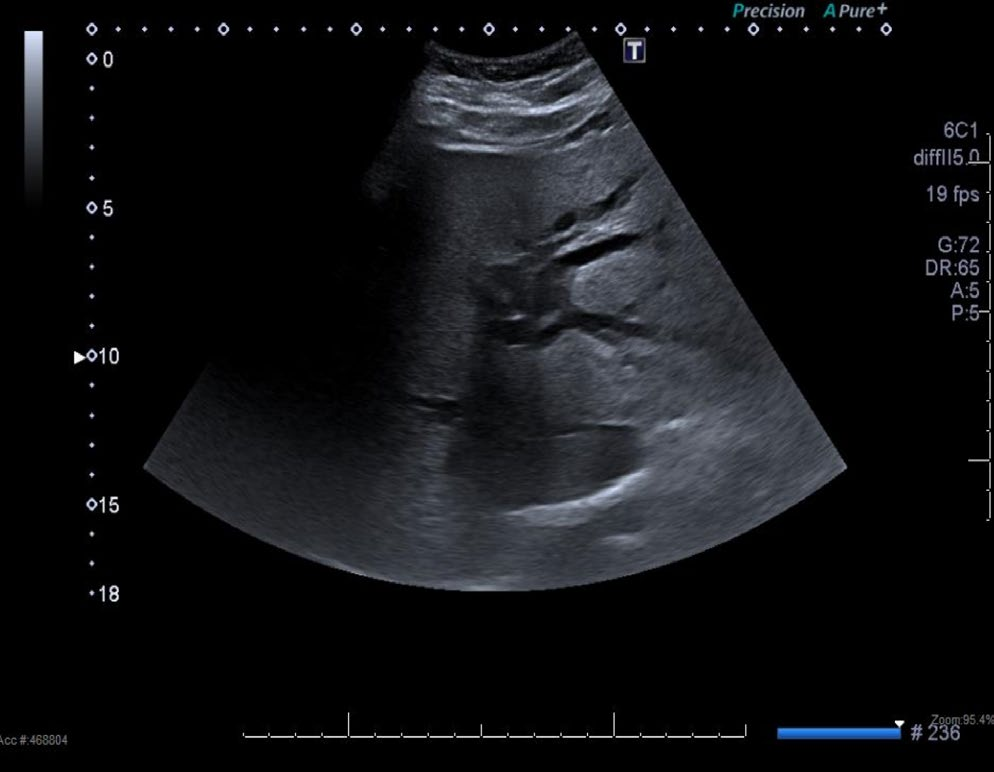
Follow‐up abdominal ultrasound demonstrating persistent dilatation of the intrahepatic bile ducts.

**FIGURE 3:**
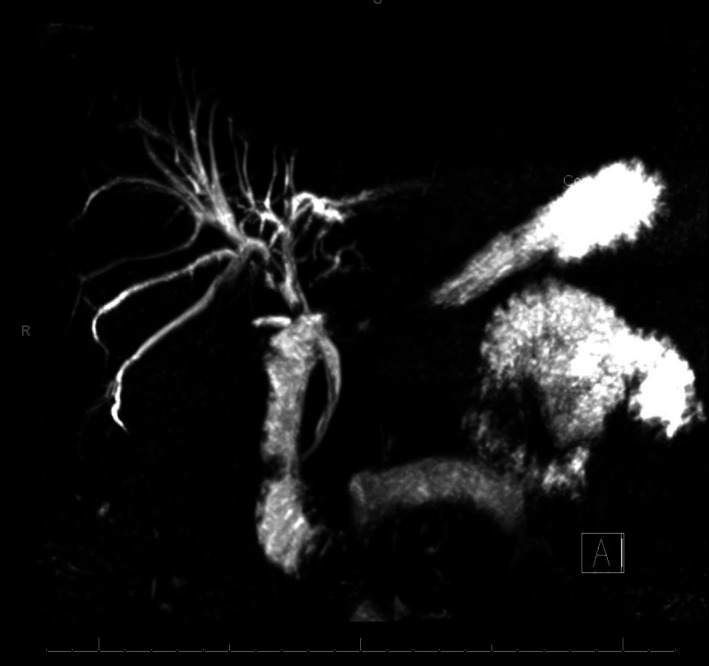
MRCP showing dilated bile ducts with multiple stones and a CBD stent.

**FIGURE 4:**
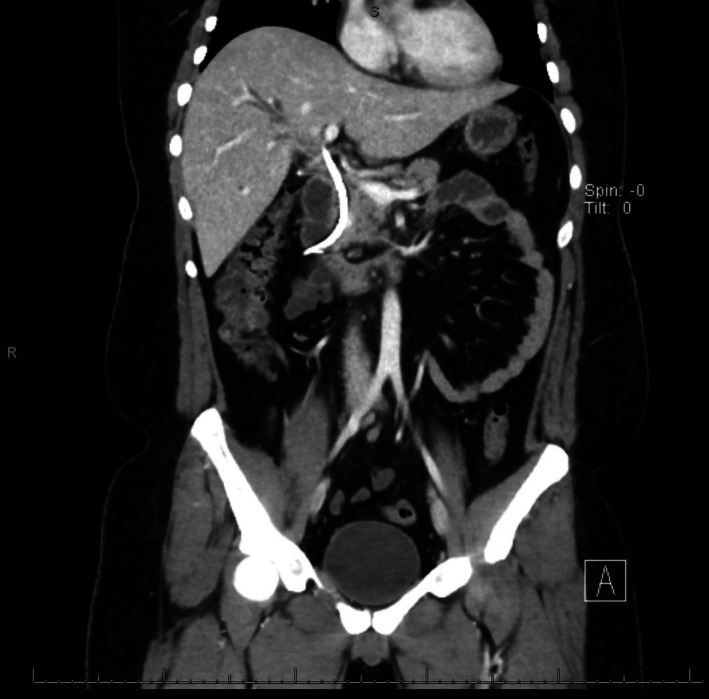
CT scan showing a CBD stent extending into the hepatic duct, with dilatation of the right hepatic duct and no visible dense stones (The image was edited by Paint 3D to hide the personal details of the patient).

Follow‐up ultrasound showed persistent intrahepatic and extrahepatic ductal dilatation with microlithiasis (Figure 2). MRCP demonstrated moderate intrahepatic and common hepatic duct dilatation with multiple intraductal stones and CBD stones adjacent to the stent, indicating ongoing cholangitis (Figure 3). CT showed the stent extending into the left hepatic duct, a dilated right hepatic duct, but no dense stones (Figure 4). One week later, repeat MRCP revealed mild residual dilatation and recurrent biliary stones, with interval improvement in cholangitis.

Subsequent imaging after the last urgent ERCP showed significant resolution of biliary dilatation. No sizable obstructive stones were found, although intraductal gas bubbles and biliary sludge persisted. The patient's LFTs when discharged were normal (ALT: 21 U/L, AST: 20 U/L, total bilirubin: 12 μmol/L, conjugated bilirubin: 6 μmol/L, and GGT: 42 U/L).

## 5 Discussion

Low phospholipid‐associated cholelithiasis (LPAC) syndrome is an uncommon but increasingly identified hereditary condition marked by the development of stones in both intrahepatic and extrahepatic bile ducts resulting from defective phospholipid excretion. This syndrome is mainly linked to pathogenic variants in the *ABCB4* gene, which encodes the MDR3 transporter responsible for the transportation of phosphatidylcholine into bile. Deficiency in this transport process enriches bile with cholesterol and bile acids lacking adequate phospholipid protection, promoting cholesterol crystallization and biliary mucosal injury. Clinically, LPAC typically presents before age 40 and recurs post‐cholecystectomy, serving as a key diagnostic clue [3].

Our case illustrates a typical clinical course characterized by recurrent episodes of cholangitis and the formation of bile duct calculi several years following cholecystectomy, even in the absence of established risk factors such as alcohol consumption or obesity. The diagnosis is often delayed due to a lack of awareness of clinicians [5].

Diagnostic imaging plays a central role, with transabdominal ultrasound revealing comet‐tail artifacts—indicative of cholesterol microlithiasis—and MRCP proving effective in detecting intrahepatic lithiasis or bile duct dilatation [2, 6].

Repeated MRCP examinations in this patient confirmed a persistent biliary blockage, necessitating multiple ERCP procedures for therapeutic management.

In the context of LPAC, ERCP functions dually as a diagnostic modality and a therapeutic measure, facilitating both stone removal and stent insertion when managing biliary obstruction or cholangitis [7].

Our patient required several sessions of ERCP, involving balloon extraction and stent placement, to control repeated occurrences of biliary sepsis. The necessity for multiple procedures before reaching a definitive diagnosis highlights a frequent diagnostic pitfall in LPAC, where symptoms are mistakenly attributed to residual choledocholithiasis rather than the disease's pathophysiological basis. Management of LPAC primarily involves UDCA therapy, which increases bile secretion, lowers cholesterol saturation in bile, and helps maintain normal function of the liver and bile ducts. Prolonged administration of UDCA significantly lowers stone recurrence and decreases reliance on repeated ERCPs [8]. In the present case, initiating UDCA treatment led to both clinical stabilization and the cessation of acute episodes. This therapeutic effect is corroborated by existing literature, which demonstrates that over 90% of patients show symptomatic improvement within weeks of starting UDCA and maintain long‐term relief. Despite advances in genetic diagnostics, mutations in the ABCB4 gene are detected in only 30%–50% of LPAC cases, thus necessitating a continued reliance on clinical evaluation and imaging for accurate diagnosis [2].

This case highlights the critical need to identify the characteristic triad comprising early‐onset biliary symptoms, recurrence following cholecystectomy, and the presence of intrahepatic microlithiasis. Without appropriate intervention, LPAC may evolve into chronic cholangitis, biliary cirrhosis, or, in rare instances, cholangiocarcinoma [8].

The clinical course of our patient is consistent with prior reports, highlighting the necessity of considering LPAC in the differential diagnosis of recurrent cholangitis and common bile duct stones, particularly when other causes have been ruled out. Although stenting provided transient symptomatic relief, durable remission was achieved only with UDCA therapy, confirming that cholecystectomy alone is inadequate for long‐term management of LPAC [1].

In conclusion, LPAC is a treatable condition that requires increased awareness among clinicians. Early identification through imaging and clinical criteria, combined with prompt initiation of UDCA and appropriate endoscopic therapy, can prevent complications and improve patient outcomes. Our case adds to the growing body of literature advocating for a comprehensive approach to LPAC, combining surveillance, medical therapy, and procedural intervention to achieve long‐term remission.

## Author Contributions

**Faris Bandar Alrashdan:** conceptualization, resources, writing – original draft, writing – review and editing. **Ramy Mahmoud Fathy Elbarody:** conceptualization, resources, writing – review and editing. **Atheer Abdullah Ali Aldra:** investigation, resources, writing – review and editing. **Mahmoud Salah Abdalhakeem Mohammed:** investigation, resources, writing – review and editing. **Abdulaziz Althafery:** investigation, visualization, writing – review and editing. **Haidara Bohsas:** methodology, writing – original draft, writing – review and editing. **Bisher Sawaf:** methodology, supervision, writing – review and editing. **Nashaat Kamal Hamdy Elkalagi:** methodology, supervision, writing – review and editing.

## Consent

Written informed consent was obtained from the patient for the publication of this case report.

## Conflicts of Interest Statement

The authors declare no conflicts of interest. The authors utilized ChatGPT (version 4) to enhance the readability and correct grammatical errors in the manuscript.

## Data Availability

Data sharing not applicable to this article as no datasets were generated or analyzed during the current study.
